# Comparative analysis of slot dimension in lingual bracket systems

**DOI:** 10.1186/1746-160X-5-27

**Published:** 2009-12-15

**Authors:** Anton Demling, Marc P Dittmer, Rainer Schwestka-Polly

**Affiliations:** 1 Department of Orthodontics, Hannover Medical School, Carl-Neuberg-Strasse 1, 30625 Hannover, Germany

## Abstract

**Background:**

Orthodontic treatment with fixed appliances requires - among others - the correct clinical expression of torque, which depends on the precise fitting of archwire and slot. Especially in the lingual technique torque problems become clinically more evident than in labial appliances also with respect to the vertical alignment of teeth due to different distances from the center of resistance. The purpose of the present study was to compare the preciseness of slot dimensions of different lingual bracket systems.

**Methods:**

Three lingual bracket systems were included in the study (7^th ^Generation and STb, Ormco, Glendora, CA, USA; Incognito, TOP-Service/3 M Unitek, Monrovia, CA, USA). Non destructive analysis of vertical slot dimensions was performed using precision pin gauges (Azurea, Belprahon, Switzerland) that were tapered in increments of 0.002 mm (0.00008 inch). The sizes of 240 incisor and canine brackets were measured per system (total: 720). Data were compared using one-way ANOVA. A p-value < 0.05 was considered statistically significant.

**Results:**

Average slot dimensions were 0.467 mm ± 0.007 mm (0.0184 inch ± 0.0003 inch) for the 7^th ^Generation bracket system, 0.466 mm ± 0.004 mm (0.0183 inch ± 0.0001) inch for the STb bracket system and 0.459 mm ± 0.004 mm (0.0181 inch ± 0.0001) inch for the Incognito bracket system. Differences between systems were statistically significant (p < 0.05).

**Conclusions:**

The analyzed bracket systems for lingual treatment exhibited significant differences in slot dimension that will clinically result in torque play. These aspects must be considered in lingual orthodontic treatment.

## Background

In recent years, an increased number of adult orthodontic patients have sought the correction of malaligned teeth. These patients are frequently focused on dental esthetics in the anterior region. For socio-cultural reasons, adult patients favor invisible orthodontic treatment over eye-catching treatment by buccal appliances. Hence, fixed lingual bracket systems have been developed over the last 30 years [1-4]. Due to the large bucco-lingual dimension of these prefabricated bracket systems, these systems were reported to cause clinical problems, such as speech dysfunction as a result of restricted functional space for the tongue, oral discomfort due to injury or irritation of the tongue, and restriction of mastication [5-7]. The implementation of customized lingual brackets and computerized archwire fabrication resulted in a decrease of subjective impairments [8-10]. Thanks to clinical simplification in using customized appliances, the indication for lingual orthodontics has been extended to adolescents [11].

**Figure 1:**
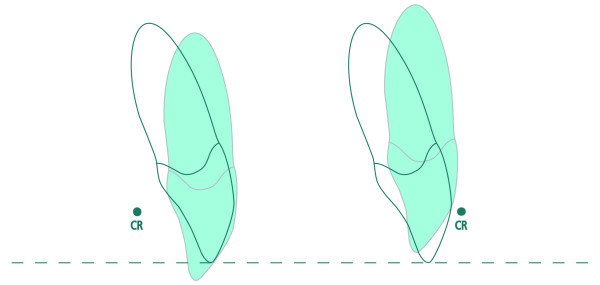
**Effects of torque loss**. Comparison of torque loss (-15 degrees) in lingual and buccal technique with respect to vertical side effects (CR = Centre of Rotation).

Besides visibility of the appliance during orthodontic treatment, the labio-lingual inclination of maxillary and mandibular incisors and canines is considered by patients and orthodontists to be an important determinant in providing esthetic outcome after orthodontic treatment. Furthermore, the correct inclination of the anterior teeth is essential in providing good occlusion in anterior and posterior regions, and is basically dependent on the correct expression of torque. This torque expression can be achieved by using slot-filling archwires or twisting and inserting undersized archwires. Nevertheless, various factors affect the correct torque expression of a preadjusted appliance [12]. These factors include material properties such as hardness and modulus of elasticity of the archwire and bracket, variations of manufacturing processes including milling and casting of brackets, as well as clinical procedures like mode of ligation [13,14]. Furthermore, a patient's individual tooth morphology also influences the clinical outcome of torque [15,16]. In the literature, it was shown that oversized slots lead to a clinically relevant torque loss [17]. Due to the longer lever arm in lingual orthodontics, slot precision must be considered as a key factor that influences tooth position not only in the labio-lingual but also in the vertical dimension [18] (see Figure 1).

Therefore, the aim of this study was to evaluate the slot precision of frequently used lingual appliances.

## Methods

**Figure 2:**
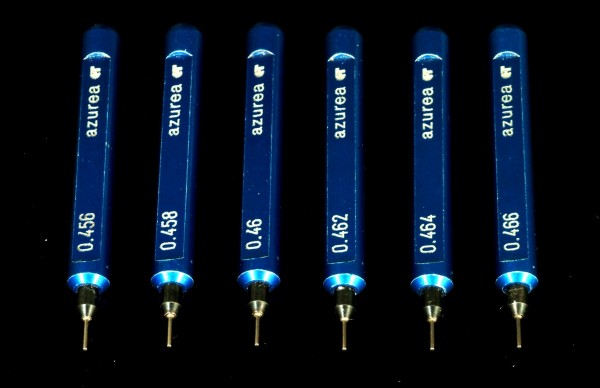
**Pin gauges**. Precision pin gauges tapered in increments of 0.002 mm (0.00008 inch).

Three lingual bracket systems were included in the study: 7^th ^Generation (Ormco, Glendora, CA, USA), STb (Ormco) and Incognito (TOP Service/3 M Unitek, Monrovia, CA, USA). All brackets were manufactured with a slot dimension of 0.457 mm (0.018) inch. For each system, the sizes of 240 incisor and canine bracket slots were measured (a total of 720). Non-destructive analysis of 0.018 inch slot dimensions was performed by one operator using precision pin gauges (Azurea, Belprahon, Switzerland). Pin gauges were tapered in increments of 0.002 mm (0.00008 inch), Figure 2.

**Figure 3:**
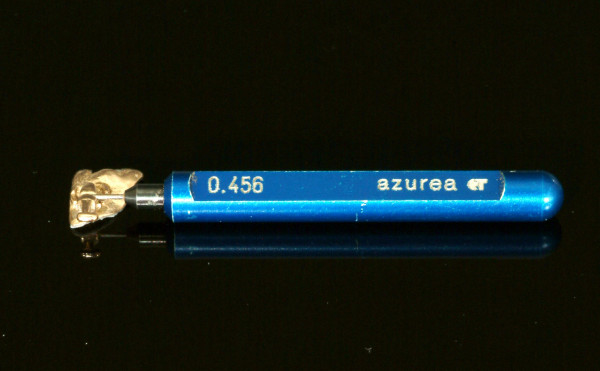
**Start of measurement**. Start of measurement of slot precision by insertion of the smallest pin gauge with a dimension of 0.456 mm (0.01795 Inch).

**Figure 4:**
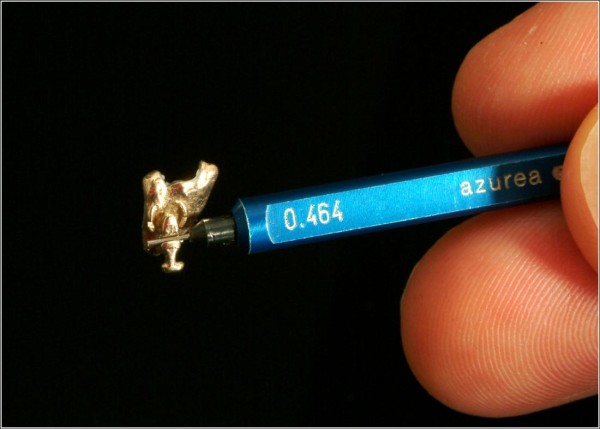
**End of measurement**. Measurement of slot precision ended when the bracket was attached to the pin gauge by friction.

Measurements of slot precision were performed starting with the insertion of the smallest pin gauge with a dimension of 0.456 mm (0.01795 inch), Figure 3. Consecutive insertion of pin gauges was continued until the bracket was attached to the pin gauge by friction (Figure 4). The value of the pin gauge one increment below the finally inserted one was documented as slot size.

Power and sample sizes were calculated using nQuery Advisor 5.0 (Statistical Solutions, Saugas, Massachusetts, USA). Power calculation revealed that the ANOVA F-test with a sample size of 240 would have a 90% power to detect a difference in means of +0.00254 mm (+0.0001 inch) and -0.00254 mm (-0.0001) inch to a reference group, assuming a within-group standard deviation of 0.01524 mm (0.0006 inch). Documentation and evaluation of the data was performed using the Statistical Package for the Social Sciences, Version 17.0 for Windows (SPSS, Chicago, Illinois, USA). The Kolmogorov-Smirnov test was applied to test for normal distribution. As data were distributed normally, they were compared using one-way ANOVA. Post-hoc testing was performed with the Fisher's LSD test. All tests were performed two-tailed with a significance level of α = 0.05.

## Results

**Figure 5:**
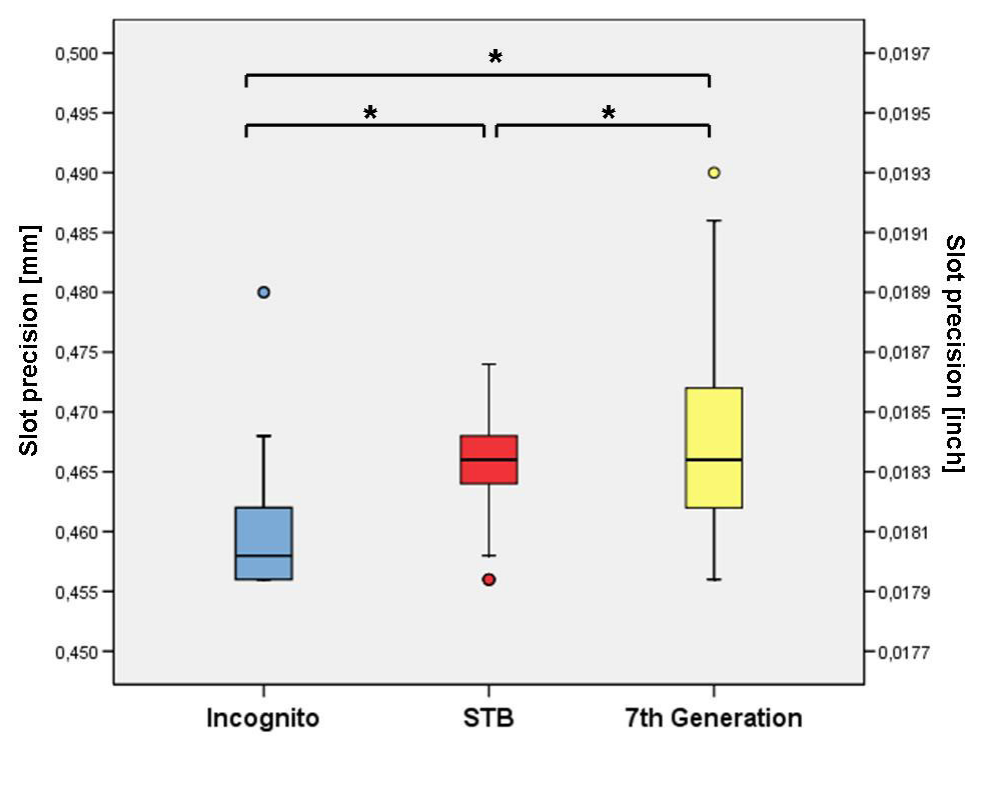
**Slot precision**. Results of determined slot precision (* = p < 0.001).

Results of experimental measurements are presented as a boxplot in Figure 5 and in Table 1. Average slot dimensions were 0.467 mm ± 0.007 mm (0.0184 inch ± 0.0003 inch) for the 7^th ^Generation bracket system, 0.466 mm ± 0.004 mm (0.0183 inch ± 0.0001 inch) for the STb bracket system and 0.459 mm ± 0.004 mm (0.0181 inch ± 0.0001 inch) for the Incognito system. The calculated medians for the included lingual bracket systems were 0.4660 mm (0.0184 inch) for the 7^th ^Generation and the STb bracket and 0.4580 mm (0.0180 inch) for the Incognito bracket. Statistical analysis revealed significant differences in slot precision between all analyzed bracket systems, with the highest precision for the Incognito bracket and the lowest for the 7^th ^Generation bracket (p < 0.001).

**Table 1:** Means, minimum and maximum values of slot precision (mm) with respect to bracket location

		Right canine (n = 40)	Right lateral incisor (n = 40)	Right central incisor (n = 40)	Left central incisor (n = 40)	Left lateral incisor (n = 40)	Left canine (n = 40)
	Mean	0.470	0.468	0.466	0.466	0.466	0.468
7th							
Generation	Min.	0.456	0.456	0.456	0.456	0.456	0.456
	
	Max.	0.484	0.480	0.480	0.486	0.490	0.486

	Mean	0.466	0.466	0.466	0.466	0.465	0.466
	
STb	Min.	0.458	0.456	0.456	0.460	0.456	0.456
	
	Max.	0.472	0.470	0.474	0.474	0.470	0.474

	Mean	0.459	0.461	0.460	0.459	0.460	0.459
	
	Min.	0.456	0.456	0.456	0.456	0.456	0.456
Incognito							
	Max.	0.466	0.466	0.480	0.468	0.466	0.466

## Discussion

The clinical expression of torque can be achieved by inserting archwires with gradually increased dimensions. However, due to undersized archwire dimensions, edge bevelling and oversized slot dimensions, a significant play of the archwire in the slot can be observed [19-21]. These factors result in a clinically relevant torque loss.

Therefore, the literature describes various methods for the determination of slot precision. For example, a metallurgic method was used to measure bracket slot height in stainless steel brackets after preparation of cross-sectional cuts through the bracket slot [22]. Afterwards, the slot height was measured to the nearest 0.01 mm with a Zeiss Axioscope. This method was characterized by destructive analysis of brackets, considerable technical effort, and low accuracy. Consequently, methods were improved by estimating effective slot height by using a formula that describes the relationship between bracket slot height, wire dimensions, wire edge bevel and torsional play [17]. This method has the advantage of high precision and non-destructive analysis, but again requires great technical effort. To allow determination of slot precision in large samples with a high degree of accuracy, measurements in the present study were performed by use of high precision pin gauges. The applied increments of 0.002 mm (0.00008 inch) can be considered as very finely graduated. The application of manual measurement furthermore allowed non-destructive analysis, and was therefore associated with low costs.

The standards for the precision of orthodontic attachments in Germany are defined in DIN 13971-2. According to this standard, a 0.0018 inch slot should have a slot width between 0.0018 inch (0.46 mm) and 0.00197 inch (0.50 mm) [23]. In the present investigation, all lingual bracket systems fulfilled these requirements, with measured average slot precisions between 0.0181 inch for the Incognito bracket and 0.0184 inch for the 7^th ^Generation bracket.

However, significant differences in slot precision were found between all investigated bracket systems. The extremely high accuracy of the Incognito bracket can be explained by the manufacturing process. All Incognito brackets undergo subsequent manual measurement of slot precision [8]. Therefore, the tolerance range of slot dimension guaranteed by the manufacturer is 0.0180 inch to 0.0183 inch.

Although the other two brackets fulfilled the DIN standard, the clinical implications of torque play must be carefully considered. The correct buccolingual inclination of maxillary teeth is particularly critical in establishing an esthetic smile line, proper anterior guidance, and a Class I canine and molar relationship [24]. One study showed that an additional anterior inclination of 5° generates 1 mm of additional arch length [25]. Consequently, the correct clinical expression of torque can be regarded as a key factor for successful orthodontic treatment, but which is mechanically jeopardized in lingual orthodontics by the longer lever arm [18]. As the Incognito bracket is a customized bracket, it has a shorter lever arm than the other two lingual bracket systems. This factor must be regarded in addition to the significant differences in slot precision when determining the clinical performance of lingual bracket systems with respect to the clinical expression of torque.

## Conclusions

In the present study, all investigated lingual bracket systems fulfilled the DIN 13971-2 standard with average slot sizes of 0.467 mm (0.0184 inch) for the 7^th ^Generation bracket system, 0.466 mm (0.0183 inch) for the STb bracket system and 0.459 mm (0.0181 inch) for the Incognito system. However, slot precision differed statistically significantly between all evaluated bracket systems.

## Competing interests

The authors declare that they have no competing interests.

## Authors' contributions

AD performed measurement of slot dimensions, statistics and wrote major parts of the manuscript. MPD assisted in performance of statistics and wrote parts of the manuscripts. RSP conceived the study design and participated in preparation of the manuscript including interpretation of data. All authors read and approved the final manuscript.
